# Evaluating the impact of a standardised pre‐operative patient management protocol on major post‐operative complications in dogs undergoing BOAS surgery

**DOI:** 10.1111/jsap.13881

**Published:** 2025-06-17

**Authors:** C. D. L. Webb, N. Gall, L. B. Meakin, E. J. Friend

**Affiliations:** ^1^ Small Animal Referral Hospital, Langford Vets University of Bristol Bristol UK; ^2^ Present address: The Royal Veterinary College Hatfield UK

## Abstract

**Objectives:**

Obstructive sleep apnoea syndrome is a common human upper respiratory tract disease that resembles Brachycephalic Obstructive Airway Syndrome in dogs, and pre‐operative lifestyle changes are often recommended to minimise perioperative risk. To assess the effect of a standardised pre‐operative protocol and early discharge on major post‐operative complications in dogs undergoing Brachycephalic Obstructive Airway Syndrome surgery.

**Materials and Methods:**

Medical records of Brachycephalic Obstructive Airway Syndrome surgery from February 2018 to May 2022 were evaluated. Exclusions comprised breeds other than the pug, French bulldog and English bulldog, patients undergoing concurrent procedures or those receiving partial pre‐operative management, resulting in 45 pre‐operative protocol cases and 45 controls. Post‐operative regurgitation and major complications (defined as a requirement for >48 hours of hospitalisation, requirement for rehospitalisation within 48 hours, post‐operative temporary/permanent tracheostomy or death) were recorded for each group.

**Results:**

Median age and body condition were not different between groups. Pre‐operative protocol patients had more severe respiratory and gastrointestinal clinical signs, were more likely to be affected by pre‐operative regurgitation and had a higher grade of laryngeal collapse compared to controls. Median hospitalization time from surgery to discharge was shorter for the pre‐operative protocol group (4.5 hours) compared to controls (29 hours). Major complications occurred in 5/45 (11.1%) pre‐operative protocol cases and 4/45 (8.9%) control cases, with no significant difference. Post‐operative regurgitation was noted in 14/45 (31.1%) pre‐operative protocol cases and 19/45 (42.2%) control cases within 24 hours of surgery.

**Clinical Significance:**

Although introducing a pre‐operative protocol for patients undergoing Brachycephalic Obstructive Airway Syndrome corrective surgery cannot currently be recommended, no detriment was observed in patients being managed in this fashion.

## INTRODUCTION

Traditional surgical techniques to alleviate upper respiratory tract (URT) obstruction, involving a combination of nasal widening, soft palate resection, tonsillectomy and laryngeal ventriculectomy, have been shown to significantly improve both respiratory and gastrointestinal clinical signs in patients with Brachycephalic Obstructive Airway Syndrome (BOAS) (Haimel & Dupré, 2015; Kaye et al., 2018; Mayhew et al., 2022; Poncet et al., 2006; Riecks et al., 2007; Seneviratne et al., 2020; Torrez & Hunt, 2006). Compared with non‐brachycephalic dogs, however, general anaesthesia of brachycephalic dogs is associated with an increased risk of post‐operative anaesthetic complications, with the most common being aspiration pneumonia (Gruenheid et al., 2018).

Recent studies have shown that dogs presented for BOAS surgery with a history of regurgitation were predisposed to post‐operative regurgitation (Costa et al., 2020; Fenner et al., 2020). The incidence of post‐operative vomiting or regurgitation was also significantly related to the incidence of respiratory complications (Lindsay et al., 2020). It follows that appropriate management of patients should involve attempts to mitigate these risks prior to BOAS corrective surgery (Downing & Gibson, 2018). The use of pre‐operative omeprazole has previously been demonstrated to reduce the rate of intra‐operative gastroesophageal reflux (Panti et al., 2009), and post‐operative use has been shown to improve the results of upper airway surgery (Poncet et al., 2006).

In humans, obstructive sleep apnoea syndrome (OSAS) is a common URT disease that appears to resemble BOAS (Downing & Gibson, 2018; Martinez & Faber, 2011). Similarly, OSAS is known to increase the incidence of gastro‐oesophageal reflux (GOR), with the latter further exacerbating symptoms of OSAS (Wu et al., 2019). The use of proton pump inhibitors has demonstrated a statistically significant reduction in apnoea attacks as early as the third week of treatment (Bortolotti et al., 2006). They are recommended for patients with concurrent OSAS and GOR, as long‐term treatment has been shown to reduce the apnoea‐hypopnea index, snoring and daytime sleepiness (Friedman et al., 2007; Orr et al., 2009). OSAS patients are also at increased risk of peri‐operative complications and will benefit from weight loss, use of continuous positive airway pressure therapy, avoiding smoke and improvement of fitness prior to anaesthesia (Martinez & Faber, 2011). This approach may therefore be of consideration for patients presenting for routine BOAS surgery (Downing & Gibson, 2018).

In this retrospective study, we compare data on the immediate post‐operative complications for dogs undergoing BOAS corrective surgery where a pre‐operative protocol (POP) was recommended to the owner and control dogs where this was not performed. We hypothesised that the incidence of complications would be lower for POP patients undergoing URT surgery compared to control patients.

## MATERIALS AND METHODS

### The pre‐operative protocol

At the authors' institution, a multi‐disciplinary small animal referral hospital in the United Kingdom, all patients referred for BOAS surgery from December 2018 were recommended to follow a POP. The reasons for introducing the POP (similar to management of pre‐operative OSAS patients) were to reduce the risks associated with surgery and anesthesia and improve the post‐operative outcomes following URT procedures.

An initial consultation was performed by a board‐certified (or residency‐trained) surgeon or ECVS resident under supervision and assisted by a senior soft‐tissue nurse. Patients were assessed for the severity of respiratory and gastrointestinal clinical signs, based upon the Poncet grading scheme (Poncet et al., 2005), and recorded in the clinical notes as mild, moderate or severe (to denote grade 1, 2 or 3, respectively).

All patients were started on a course of proton pump inhibitors (Omeprazole, 1 mg/kg twice a day), and clients were counselled in monitoring for reflux, regurgitation and respiratory distress. Lifestyle changes were aimed at minimising stress, avoiding overheating, and exposure to smoke. Transition to a hypoallergenic diet, slow feeding and feeding from a step with post‐meal elevation were encouraged. Finally, a long‐term goal of reducing body condition score (BCS) was set at 4/9 (Laflamme, 1997). Written guidelines were provided for each client to reinforce these points (see Supporting Information), and surgery was planned for 4 to 8 weeks after the initial consultation. It is important to note that lack of improvement was not considered essential for surgery to be performed. Although a second Poncet grade was not recorded, if there was notable subjective improvement in a patient's symptoms within this period, then surgery would be cancelled.

Patients were admitted on the day of, or the day before, surgery. General anaesthesia was induced and maintained using standard protocols for the institution. Most patients were premedicated with dexmedetomidine (1 to 3 mcg/kg Dexdomitor 0.5 mg/mL; Vetoquinol, UK) in combination with methadone (0.2 mg/kg Comfortan 10 mg/mL; Dechra, UK), induced using propofol (1 to 4 mg/kg PropoFlo Plus 10 mg/mL; Zoetis, UK) to effect, and were maintained on inhaled sevoflurane (2% to 3% SevoFlo 100% w/w, Zoetis, UK) in 100% oxygen. The grade of laryngeal collapse was recorded as none, mild, moderate or severe, according to previously defined criteria (Torrez & Hunt, 2006). Surgery was performed or supervised by a board‐certified (or residency‐trained) surgeon using a combination of techniques of their preference. Recovery was in the intensive care ward for all patients, and routine patient discharge was arranged for the same day as surgery. Follow‐up phone calls were performed 1 day, 1 week and 1 month after surgery. Any regurgitation identified by the carer following discharge was recorded on the patient file. Where the POP was declined, patients were taken to surgery within 24 hours.

Prior to the introduction of the POP, medical treatments for gastroesophageal reflux and lifestyle changes were inconsistent. Patients would typically present for initial assessment the day before surgery. Anaesthetic protocols and surgical techniques were the same as for POP‐managed patients. Recovery was similarly in the intensive care ward for all cases, but in most instances, routine patient discharge was the following day (according to the standard protocol in place at the time).

### Medical records

Electronic records were retrospectively reviewed for patients undergoing URT surgery for BOAS. Specifically, this was performed by manually screening theatre bookings to identify cases. The aim was to capture all surgical patients receiving the POP and an equal number of historic control patients.

Patients were eligible for inclusion in the POP group if the standardised pre‐operative medical management and lifestyle protocol was recommended, and owners agreed to delay surgery to implement these changes. Patients were assigned to the control group if no pre‐operative management was employed, and surgery was performed within 24 hours of initial assessment. Patients were excluded if there was partial medical and lifestyle management before establishment of the POP, any concurrent procedures were performed at the time of URT surgery, if there were inadequate medical records and follow‐up, or if patients were a breed other than French bulldog, pug or English bulldog.

Recorded information included signalment, date of initial assessment and surgery, history of regurgitation, a subjective severity of respiratory and gastrointestinal clinical signs at the time of initial assessment, duration of pre‐operative medical management, age at surgery, weight and BCS at surgery, grade of laryngeal collapse, surgery performed, duration of hospitalisation, incidence of post‐operative regurgitation within 24 hours and the incidence of major complications.

Major complications were defined as a requirement for >48 hours of hospitalisation for supportive care or a requirement for rehospitalisation within 48 hours (as a direct result of URT surgery), post‐operative temporary/permanent tracheostomy, or death (modified from the criteria used in a study by Tarricone et al., 2019).

### Statistical analysis

Statistical analysis was performed using IBM SPSS Version 26.0 (IBM Corp. Released 2019. IBM SPSS Statistics for Windows, Version 26.0. Armonk, NY: IBM Corp). POP and control groups were compared to ordinal variables using the Mann–Whitney *U* test; when independent variables included more than two groups, a Kruskal–Wallis test was used to assess against ordinal variables. Independent scale variables were compared to ordinal variables or non‐normally distributed scale variables using a Spearman's rank correlation test. By convention, a P‐value of ≤0.05 was considered statistically significant.

### Study design

This study was approved by the University of Bristol and Animal Welfare Ethical Review Body with the unique code VIN/20/011.

## RESULTS

One hundred and forty‐seven cases were identified over a 52‐month period from February 2018 to May 2022. Seventy‐five of these were POP cases between December 2018 and May 2022. The remaining 72 were control cases from February 2018 onwards – this included four cases after December 2018 where the POP was declined. These patients were then taken to surgery the following day without the chance to instigate pre‐operative management changes.

Based upon breed, two bulldog crossbreeds and one Chow were excluded from the POP group. Twenty‐seven patients were also excluded from further analysis as they underwent concurrent procedures during the same anaesthetic. Nineteen of these excluded patients underwent an additional neutering procedure, six patients were treated surgically for ophthalmic conditions, one patient underwent hiatal hernia repair, and one patient underwent both neutering and ophthalmic procedures.

In the control group, one Boston terrier and one Staffordshire bull terrier were excluded based upon breed. Fifteen patients were excluded as they received partial pre‐operative management (three of these patients were also neutered under the same anaesthetic). Ten patients were excluded as they underwent concurrent procedures during the same anaesthetic. Two of these patients underwent an additional neutering procedure, one patient had additional surgery to excise a cutaneous mass from the left flank, four patients were treated surgically for ophthalmic conditions, two patients underwent hiatal hernia repair (one of which was additionally neutered), and an MRI was performed in one patient for investigation of pelvic limb ataxia. Exclusions are summarised in Table 1.

**Table 1:** Exclusions

Reason for exclusion	POP	Control
Breed (other than pug, French bulldog or English bulldog)	3	2
Neutering	19	2
Ophthalmic surgery	6	4
Ophthalmic surgery and neutering	1	
Hiatal hernia repair	1	1
Hiatal hernia repair and neutering		1
Mass excision		1
MRI		1
Partial pre‐operative management		12
Partial pre‐operative management and neutering		3
Total exclusions	30	27

MRI Magnetic resonance imaging; POP Pre‐operative protocol

All patients were considered to have adequate medical records for evaluation. As a result, 45 POP cases and 45 control cases were retained for further analysis. In the POP group, there were 27 French bulldogs, seven pugs and 11 bulldogs. Control dogs comprised 20 French bulldogs, 20 pugs and five bulldogs. Median age was 2.17 years (range 1.00 to 7.39) and 2.45 years (range 0.49 to 9.44 years) for POP and control dogs, respectively. The difference in ages was not significantly different (P = 0.91).POP dogs had a median weight of 12.5 kg (range 6 to 30.6 kg) and a median BCS of 5/9 (range 3 to 8). Control dogs had a median weight of 10 kg (range 5.6 to 40.5 kg) and a median BCS of 5/9 (range 3 to 9). The difference between body weight was significant (P = 0.013), but BCS was similar (P = 0.11) between groups. History of regurgitation and the severity of respiratory and gastrointestinal clinical signs are outlined in Table 2. 44/45 (97.8%) POP patients and 24/45 (53.3%) control patients had a history of regurgitation, which was significantly different between groups (P < 0.001). At the time of initial assessment, the median severity of respiratory clinical signs was moderate (Poncet grade 2) for the POP group and mild (Poncet grade 1) for the control group. The median grade of gastrointestinal clinical signs at this assessment was moderate (Poncet grade 2) for the POP group and mild (Poncet grade 1) for the control group. The differences in severity of respiratory and gastrointestinal clinical signs were both significantly different between groups (P < 0.001). POP cases were managed pre‐operatively for a median duration of 67 days (range 18 to 263 days) with the combination of changes described previously. Control cases were assessed the day before (*n* = 40) or on the day of (*n* = 5) surgery and had no pre‐operative management.The grade of laryngeal collapse for dogs in the POP group and control group is also shown in Table 2. There was a significantly higher degree of laryngeal collapse in the POP dogs (P = 0.006). The anaesthetic record was not attached to the electronic file in 1/45 POP cases. The anaesthetic medications for remaining patients are summarised in Table 3. The median general anaesthesia time was 65 minutes for POP dogs and 50 minutes for control dogs (P = 0.001).

**Table 2:** Clinical signs and grade of laryngeal collapse

Pre‐operative findings	Severity^†^	POP	Control	P‐value
Respiratory clinical signs^‡^	Mild	9	25	
	Moderate	20	17	<0.001*
	Severe	16	3	
Gastrointestinal clinical signs^§^	Mild	18	37	
	Moderate	17	7	<0.001*
	Severe	10	1	
	History of regurgitation	44	24	<0.001*
Laryngeal collapse	None	0	3	
	Mild	12	21	
	Moderate	28	13	
	Severe	3	3	0.006*
	Not recorded	2	5	

POP Pre‐operative protocol

^†^ Severity based on frequency of different clinical signs (Poncet et al., 2005)

^‡^ Respiratory clinical signs defined as snoring, inspiratory efforts, stress or exercise intolerance or syncope (Poncet et al., 2005)

^§^ Gastrointestinal clinical signs defined as ptyalism, regurgitation or vomiting (Poncet et al., 2005)

* Statistically significant

**Table 3:** Anaesthesia protocol

Anaesthetic drugs used	POP	Control	P‐value
Dexmedetomidine	40	40	
ACP	2	1	
Dexmedetomidine and ACP	1	1	
Ketamine and midazolam	1		
No sedative		3	
Methadone	36	37	
Fentanyl	1		
Buprenorphine	6	8	
Ketamine without opioid	1		
Propofol	34	35	
Alfaxalone	10	10	
Isoflurane	4	12	
Sevoflurane	39	33	
Desflurane	1		
Median anaesthetic duration in minutes (range)	65 (30 to 135)	50 (20 to 120)	0.001*

POP Pre‐operative protocol

* Statistically significant

Upper airway surgeries included staphylectomy (45/45 POP cases, 45/45 control cases), lateral alarplasty (40/45 POP cases, 41/45 control cases), tonsillectomy (39/45 POP cases, 9/45 control cases) and laryngeal ventriculectomy (4/45 POP cases, 15/45 control cases). The median number of upper airway surgeries performed was three and two for POP and control groups, respectively. POP dogs had significantly more upper airway surgeries performed than control dogs (P < 0.001) and there was a significant difference in the number of tonsillectomies (P < 0.001) and laryngeal ventriculectomies (P = 0.005) between groups (Table 4).

**Table 4:** Surgical procedures

Surgical procedure	POP	Control	P
Staphylectomy	45	45	–
Lateral alarplasty	40	41	–
Tonsillectomy	39	9	<0.001*
Laryngeal ventriculectomy	4	15	0.005*
Median number of URT surgeries	3	2	<0.001*

POP Pre‐operative protocol, URT Upper respiratory tract

* Statistically significant.

Median hospitalisation time from surgery to discharge was 4.5 hours (range 2.5 to 7 hours) for the POP group and 29 hours (range 6.5 to 150 hours) for the control group. The difference between the groups was significant (P < 0.001). Only two patients in the control group were discharged on the day of surgery.

### Complications

Post‐operative regurgitation occurred in 14/45 (31.1%) POP cases and 19/45 (42.2%) control cases within 24 hours of surgery, but this difference was not significant (P = 0.28).

Major complications were seen in 5/45 (11.1%) POP cases and 4/45 (8.9%) control cases (Table 5). Four out of five POP cases and all control cases with major complications also demonstrated post‐operative regurgitation. The difference in major complications between groups was not statistically significant (P = 0.72).

**Table 5:** Post‐operative complications

Complication	POP	Control	P
Postoperative regurgitation	14	19	0.28
Prolonged hospitalisation	3	4	
Tracheostomy placement		1	
Death	2		
Patients with a major complication	5	4	0.72

POP Pre‐operative protocol

Prolonged hospitalisation was required in 3/45 (6.7%) POP cases (ongoing reflux requiring intravenous anti‐emetics and fluids *n* = 3) and 4/45 (8.1%) control cases (treatment for ongoing reflux *n* = 3, tracheostomy tube management *n* = 1). In all these POP cases, discharge was performed on the day of surgery, but patients were re‐hospitalised at their primary care practice within 24 hours.

Temporary tracheostomy tube was not used in any POP case and was placed in 1/45 (2.2%) control cases for emergency management of URT obstruction (survived to discharge).

Death occurred in 2/45 (4.4%) POP cases after acute reflux and respiratory obstruction during travel back home in the car in one case, and the same was suspected to have occurred at home overnight in the other case. None of the control cases died within 24 hours of surgery.

## DISCUSSION

Our study evaluates the immediate post‐operative complications of French bulldogs, pugs and English bulldogs undergoing traditional URT surgery for BOAS, following the introduction of a standardised POP. These patients were discharged a median duration of 4.5 hours following surgery. The primary aims of the POP were to decrease peri‐operative risk and to improve long‐term results. No difference in the rate of post‐operative regurgitation or major complications was identified between the POP group and controls, and the alternative hypothesis is therefore rejected.

Upper gastrointestinal disease is seen commonly in human patients with OSAS, and antacids are considered beneficial (Bortolotti et al., 2006; Friedman et al., 2007; Orr et al., 2009; Wu et al., 2019). Upper gastrointestinal tract lesions are also a very frequent finding in BOAS‐affected dogs (Poncet et al., 2005), and 1 mg/kg omeprazole twice a day was prescribed to all POP patients for control of gastrointestinal clinical signs and to minimise the consequences of gastro‐oesophageal reflux (Marks et al., 2018; Poncet et al., 2006). To limit exacerbation of pre‐existing gastrointestinal disease, a hypoallergenic diet was recommended for all POP cases (Kook et al., 2014). Additionally, slow feeding, postural (step) feeding and post‐feeding elevations were encouraged to minimise any reflux associated with delayed oesophageal motility (Reeve et al., 2017). A history of regurgitation predisposes to postoperative regurgitation in patients presenting for BOAS surgery (Costa et al., 2020; Fenner et al., 2020), and prior regurgitation episodes were significantly more common for patients in the POP group (P < 0.001). Despite this, there was no significant difference in the occurrence of immediate post‐operative regurgitation between groups (P = 0.26).

Importantly, however, the true frequency of post‐operative regurgitation in the first 24 hours is very likely to be underestimated for the POP group who were all discharged on the day of surgery. In most cases, it was not possible to determine from the records whether an episode comprised silent regurgitation or true regurgitation. POP cases were not supervised by trained professionals following discharge, who could have otherwise identified and documented additional instances of silent regurgitation, and continuous overnight monitoring was not anticipated. More regurgitation events could therefore have been missed for the POP group and may have biased any observed benefits for this group.

The period of POP patient treatment spanned the lockdown periods of the COVID‐19 (C‐19) pandemic. It is suspected that this contributed to the increased severity of clinical signs and laryngeal collapse grade within this group, due to delays in seeking veterinary attention (Owczarczak‐Garstecka et al., 2022). These are additional confounding factors when comparing the two groups. The C‐19 pandemic may also explain the large range in the duration of medical management of POP cases, despite a recommendation for 4 to 8 weeks.

Several patients presenting for BOAS assessment prior to the introduction of the standardised POP had partial pre‐operative management, but recommendations for the duration of treatment were not well defined. It was the appreciation of the clinicians involved that there was subjective improvement in patient clinical signs by 4 to 8 weeks, hence the recommendation for the POP. It is reasonable to expect that a longer duration of management gives the opportunity for greater weight loss, greater reduction in pharyngeal oedema, and better patient acceptance of a new diet prior to anaesthesia. There may be value in the repeat assessment of Poncet grade for clinical signs on a 4‐weekly basis to determine the optimum timeline for improvement. Addition of functional respiratory grading using an exercise test (Riggs et al., 2019) or whole‐body barometric plethysmography (Liu et al., 2016) would also be of particular interest as these could provide more objective measures of change.

Concurrent non‐airway procedures increase the anaesthetic duration and (particularly intra‐abdominal surgeries) have been identified as risk factors for regurgitation (De Miguel Garcia et al., 2013; Galatos & Raptopoulos, 1995) and a negative outcome following surgical treatment for BOAS (Tarricone et al., 2019). Many exclusions (27/30 POP treated patients and 13/27 control patients) were due to concurrent procedures. The reasons for performing these procedures at the same time as URT surgery could not be identified from the records, but may include clinician experience, perceived level of importance of the concurrent procedure, or other client factors such as reduced total cost or number of visits. The practice of concurrent surgery is discouraged by the Brachycephalic Working Group, as the recommended approach would be for URT surgery to be performed first as a sole procedure, to reduce anaesthetic duration (Ladlow et al., 2021). Subsequent post‐anaesthetic risk will then be significantly decreased (Doyle et al., 2020) and incorporation of this recommendation with the POP could be of benefit for future cases. More surgeries were performed in the POP group with a median of three surgical procedures, compared to two in the control group, and is the likely reason for significantly longer anaesthesia times for POP cases.

Two dogs in the POP group and three control dogs had a previous history of BOAS surgery. There is disagreement over this factor being associated with a risk of negative outcome (Ree et al., 2016; Tarricone et al., 2019) and cases were therefore included.

Early mobilisation of post‐operative patients has been suggested to improve overall lung function (Downing & Gibson, 2018), and avoiding extended hospitalisation may reduce stress or fear‐induced tachypnoea and subsequent exacerbation of airway inflammation or collapse (Grubb, 2010). It is for these reasons that we currently aim to discharge our patients on the day of surgery and that earlier patient discharge occurred in the POP group. This is supported by recent studies wherein similar post‐operative protocols have been employed (Camarasa et al., 2023; Holloway et al., 2022). It is likely that there will always be a compromise in optimal timing for discharge; however, as the ability to immediately secure the airway is not possible once a patient has left the hospital. Indeed, two POP cases died following suspected episodes of acute reflux after being discharged on the day of surgery, and it is possible that longer hospitalisation for these patients could have improved their outcome. The authors would still favour early discharge from the hospital for patients with uncomplicated recoveries.

Further limitations of this study include the use of historic controls, which introduces bias caused by variations over time. This can include changes to surgical or anaesthetic preference, surgical experience and individual technique and recovery protocol. There was a difference in tonsillectomy and laryngeal ventriculectomy between groups, which could have resulted from these changes. Overall, the variability between groups makes it challenging to draw reliable conclusions regarding the impact of the POP.

Introducing a POP for patients undergoing BOAS corrective surgery provides an opportunity to maximise patient health before general anesthesia. Overall, there were several differences in risk factors for post‐operative complications between groups, and a benefit of combining a POP with same‐day discharge could therefore not be demonstrated. Although this management strategy cannot currently be recommended, no detriment was observed in patients being managed in this fashion.

### Author contributions

**C. D. L. Webb:** Data curation (equal); formal analysis (supporting); methodology (lead); visualization (lead); writing – original draft (lead); writing – review and editing (lead). **N. Gall:** Data curation (equal); formal analysis (lead); writing – original draft (supporting); writing – review and editing (supporting). **L. B. Meakin:** Formal analysis (supporting); software (lead); supervision (supporting). **E. J. Friend:** Conceptualization (lead); formal analysis (supporting); methodology (supporting); supervision (lead); writing – review and editing (supporting).

### Conflict of interest

None declared.

## Supporting information


Data S1.

## Data Availability

The data that support the findings of this study are available from the corresponding author upon reasonable request.
