# Supplementary material for: Evaluating the impact of a standardised pre‐operative patient management protocol on major post‐operative complications in dogs undergoing BOAS surgery
Source: J Small Anim Pract. 2025 Jun 17;66(10):725–31. doi: 10.1111/jsap.13881 (PMC12489528; doi:10.1111/jsap.13881)
Supplement: Supplementary file 1 — Data S1. [file JSAP-66-725-s001.docx]

**Brachycephalic Management**

Brachycephalic breeds have obstruction to their breathing from excessive soft tissues in their nose and throats, leading to reduced ability to exercise or cope in hot weather.

They also have reflux problems, which can worsen their ability to breath.

All brachycephalics should be managed, as outlined below, prior to surgery, as this should improve safety of the anaesthetic.

**FEEDING MANAGEMENT INSTRUCTIONS**

1. Hypoallergenic diet
   - A very bland diet is necessary as they are prone to reflux.
   - No human foods, table scraps, milk, chews, treats etc. If you wish to reward your dog, a single piece of their own kibble food can be given.
   - Veterinary prescription diets are good, and you can ask your vet about this if you like, but they tend to be expensive for long term use.
   - A good brand of commercially available hypoallergenic diet, bought from a good pet store, is good enough for most brachycephalics and will be cheaper. I tend to find the fish-based ones work best, but you’ll need to trial different ones as they are all individuals!
   - James Wellbeloved Coley and Rice or Wafcol Salmon and Potato are good examples.
   - Change over slowly, over about a week, by gradually feeding more of the new diet and less of the old.
2. A kibble food is fine, but this should be soaked in water before feeding, and should be the consistency of tinned food when cut with a knife.
   - Pouring on boiling water and allowing to cool for half an hour works well, or soak in cold water overnight.
3. Feed slowly – a ‘slow feed’ bowl is useful; alternatively putting half a house brick in their food bowl was the old-fashioned alternative! You could also slowly hand feed, but this is very time consuming.
4. Feed from a step – the front legs need to be higher than the back legs when they are eating. This could be from a low chair, a step or feeding steps can be purchased (such as the ‘Willowstep’)
5. Elevate afterwards – they should be stood up on their back legs for 2-5 minutes after eating; alternatively, they can be held upright for the same amount of time, rather like winding a baby. If you can, it may be beneficial in holding them like this at other times of the day as well.
6. Antacid medications – we use these once the swallowing study has been performed, before and after any surgery. Some dogs require these either intermittently or continuously for life, but the ‘natural’ remedies outline above, if performed properly and regularly, should reduce the reliance on medications.

**GENERAL MANAGEMENT**

1. Keep cool
   - This may mean showering/hosing down regularly in warmer weather, before and after walks.
   - Using bottled water to soak the coat during a walk.
   - Use of cool mats for the floor placed in a dog travel cage.
   - Avoid exercise on hot days, or at least go out first thing in the morning and last thing at night when the temperatures are lower.
   - Often there is a vicious cycle over several hours or days of overheating, so start these strategies early *before* they overheat.
2. Avoid excessive exercise – unfortunately, it is unrealiustic to expect any brachycephalic dog to exercise as well as a Labrador, although some can get pretty close!
3. Keep very, very slim. The ribs should be visible in brachycephalic dogs. They will always breathe better if dieted.
4. Avoid smoke – all smoking or vaping indoors should be avoided in the household; even one cigarette in the house, even in a different room or near a window, is enough to worsen their problem.
5. General health – keep their vaccinations up to date, and ensure they are wormed effectively for lungworm (check with your own vet).
6. Stressful events will often cause a ‘flare up’ of signs of reflux (and also of general brachycephalic breathing problems). Examples of these type of events are long car journeys; staying at boarding kennels or away from home; visits to the groomer or vet; sedation or anaesthetics; bringing a new animal or person into the home. If these events are anticipated, it is often worth stepping up the management for 2 weeks before and 3 to 4 weeks after the event and consider adding in antacid medication during this time as well (if already being used, the dose may be increased with guidance from your vet or additional medication can be used).

Team members involved in the care:

Clinician:

Nurse/s:
